# Fatal Adverse Events Associated With Immune Checkpoint Inhibitors in Non–small Cell Lung Cancer: A Systematic Review and Meta-Analysis

**DOI:** 10.3389/fmed.2021.627089

**Published:** 2021-02-15

**Authors:** Xiaolin Yu, Xiaomei Zhang, Ting Yao, Ye Zhang, Yanxia Zhang

**Affiliations:** ^1^Graduate School, Beijing University of Chinese Medicine, Beijing, China; ^2^Department of Respiratory, Dongfang Hospital, Beijing University of Chinese Medicine, Beijing, China; ^3^The 2nd Department of Pulmonary Disease in Traditional Chinese Medicine (TCM), China-Japan Friendship Hospital, Beijing, China; ^4^Department of Personnel and Epidemiology, Dongfang Hospital, Beijing University of Chinese Medicine, Beijing, China

**Keywords:** immune checkpoint inhibitors, fatal adverse event, non-small cell lung cancer, incidence, meta-analysis

## Abstract

**Background:** Immune checkpoint inhibitors (ICIs) have previously been reported to have a promising potential in terms of the improvement of outcomes in non–small cell lung cancer (NSCLC). Fatal adverse events (FAEs) of ICIs are relatively uncommon, and the incidence and risk in NSCLC remain unclear. In the present study, we conducted a systematic review and meta-analysis to evaluate the risk of FAEs in NSCLC patients administered with ICIs.

**Methods:** Potentially relevant studies were identified in PubMed, EMBASE, and Cochrane library database from inception to September 16, 2020. The systematic review and meta-analysis included randomized controlled trials that reported treatment-related FAEs in NSCLC. The pooled incidence and risk ratios (RRs) were calculated to evaluate prospective risk.

**Results:** Twenty clinical trials that included a total of 13,483 patients were selected for the meta-analysis. The overall incidence of FAEs was 0.65% [95% confidence interval (CI) = 0.31–1.07, *I*^2^ = 50.2%] in ICI monotherapy, 1.17% (95% CI = 0.74–1.69, *I*^2^ = 56.3%) in chemotherapy, and 2.01% (95% CI = 1.42–2.69, *I*^2^ = 5.9%) in the combination therapy (ICI and chemotherapy). ICI monotherapy was associated with lower incidence of FAEs caused by blood system disorders (RR = 0.23, 95% CI = 0.07–0.73, *P* = 0.013, *I*^2^ = 0%) and infectious diseases (RR = 0.29, 95% CI = 0.13–0.63, *P* = 0.002, *I*^2^ = 0%). The incidence of pneumonitis significantly increased in immunotherapy (RR = 5.72, 95% CI = 1.14–28.80, *P* = 0.03, *I*^2^ = 0%).

**Conclusions:** The results of the present study demonstrate that ICI monotherapy decreases the risk of FAEs, whereas the combined regimens with chemotherapy have the opposite tendency as compared to conventional chemotherapy. While the patients who received chemotherapy suffered the risks of death mainly from myelosuppression and infection, those who received immunotherapy were mainly threatened by immune-related pneumonitis.

## Introduction

Lung cancer is a major threat to human health (1). Non–small cell lung cancer (NSCLC) is the most common pathological type of lung cancer and accounts for ~85% of all lung cancer cases (2). A considerable number of patients are characterized as NSCLC with locally advanced disease (3). In the past decades, chemotherapy is the primary treatment option for advanced NSCLC; however, patients with advanced NSCLC still have a poor prognosis (4, 5). Although new therapeutic agents, like the molecular targeted therapy for lung cancer, have significantly improved the treatment of NSCLC (6), only the subset of patients with corresponding genetic mutations can benefit from this therapy (7).

Recent advances in immune checkpoint inhibitors (ICIs), which have been approved by US Food and Drug Administration for the application in the advanced NSCLC (8, 9), have made a dramatic breakthrough in the field of cancer treatment. To date, the ICI drugs have mainly included cytotoxic T-lymphocyte–associated protein 4 (CTLA-4) and programmed death-1/ligand-1 (PD-1/PD-L1) inhibitors, which could enhance the T-cell immune response to avoid the immune escape of tumor cells (10). Previous research has demonstrated a promising potential of immunotherapy in terms of improvement of clinical outcomes in advanced NSCLC (11). However, with the increased application of ICI drugs in NSCLC, the number of reports about toxicity has also increased, which warrants further research, particularly with regard to treatment-related fatal adverse events (FAEs) (12, 13). Of note, most previous studies focused on the overall incidence of adverse events among all applicable cancers or considered the specific incidence of a certain system (14–17). There are scarce reports focused on FAEs, and further investigations are needed.

In this context, to comprehensively evaluate the risk of FAEs associated with ICI drugs in NSCLC and to provide more references for the clinical management, in the present study, we conducted a systematic review and meta-analysis of published clinical trials.

## Materials and Methods

### Data Source and Search Strategy

This study was reported according to the PRISMA (Preferred Reporting Items for Systematic Reviews and Meta-Analyses) statement (18) (Supplementary Material 1). The protocol was registered in PROSPERO (Supplementary Material 2). Potentially eligible studies were identified in PubMed, EMBASE, and Cochrane library database from inception to September 16, 2020. Conference abstracts from annual meetings of the American Society of Clinical Oncology and European Society for Medical Oncology in the years 2010–2020 were also searched. During the search for relevant studies, the following key words were used: “Nivolumab,” “Pembrolizumab,” “Atezolizumab,” “Durvalumab,” “Avelumab,” “Ipilimumab,” “Tremelimumab,” “PD-1,” “PD-L1,” “CTLA-4,” “non–small cell lung cancer.” Further detail on the search strategy is provided in Supplementary Table 1.

### Inclusion and Exclusion Criteria

The final dataset included previously published studies that met the following five inclusion criteria: [1] participants: patients histologically diagnosed as NSCLC; [2] intervention: ICI monotherapy alone or combined with immunotherapy, chemotherapy, target therapy, and radiotherapy; [3] comparison: the independent control arm administered with chemotherapy; [4] outcomes: reporting the treatment-related FAEs; [5] randomized controlled trials (RCTs). Exclusion criteria used in the present meta-review were as follows: [1] reviews and quality of life studies, [2] animal studies or basic experiments, [3] single arm trials, and [4] non-English articles.

### Data Extraction and Quality Assessment

Two individual reviewers independently extracted the data according to a self-designed collection form. Disagreements were resolved through discussion and consensus among all authors. From the included studies, we extracted the following information: name of the first author, year of publication, study name, registered clinical trial ID, study phase, sample size, treatment regimens, and treatment-related FAEs. The methodological quality of the reviewed studies was evaluated using the Cochrane Risk-of-Bias Tool (19).

### Statistical Analyses

The clinical heterogeneity of the studies included in the review was evaluated by the study design, characteristics of patients, interventions, and outcomes. The meta-analysis was conducted using the meta package in R software. Pooled risk ratios (RRs) and 95% confidence intervals (CIs) were calculated to evaluate the prospective risk. In order to avoid overestimation caused by the continuity correction for zero events, the pooled proportion was calculated using Freeman-Tukey double-arcsine transformation. The heterogeneity was assessed by the *I*^2^ and Cochrane Q statistic. *I*^2^ > 50% was considered to represent significant heterogeneity, and a random-effects model was selected. Otherwise, a fixed-effects model was conducted. The publication bias was evaluated by the funnel plot and Egger's linear regression test.

## Results

### Search Results

**Figure 1:**
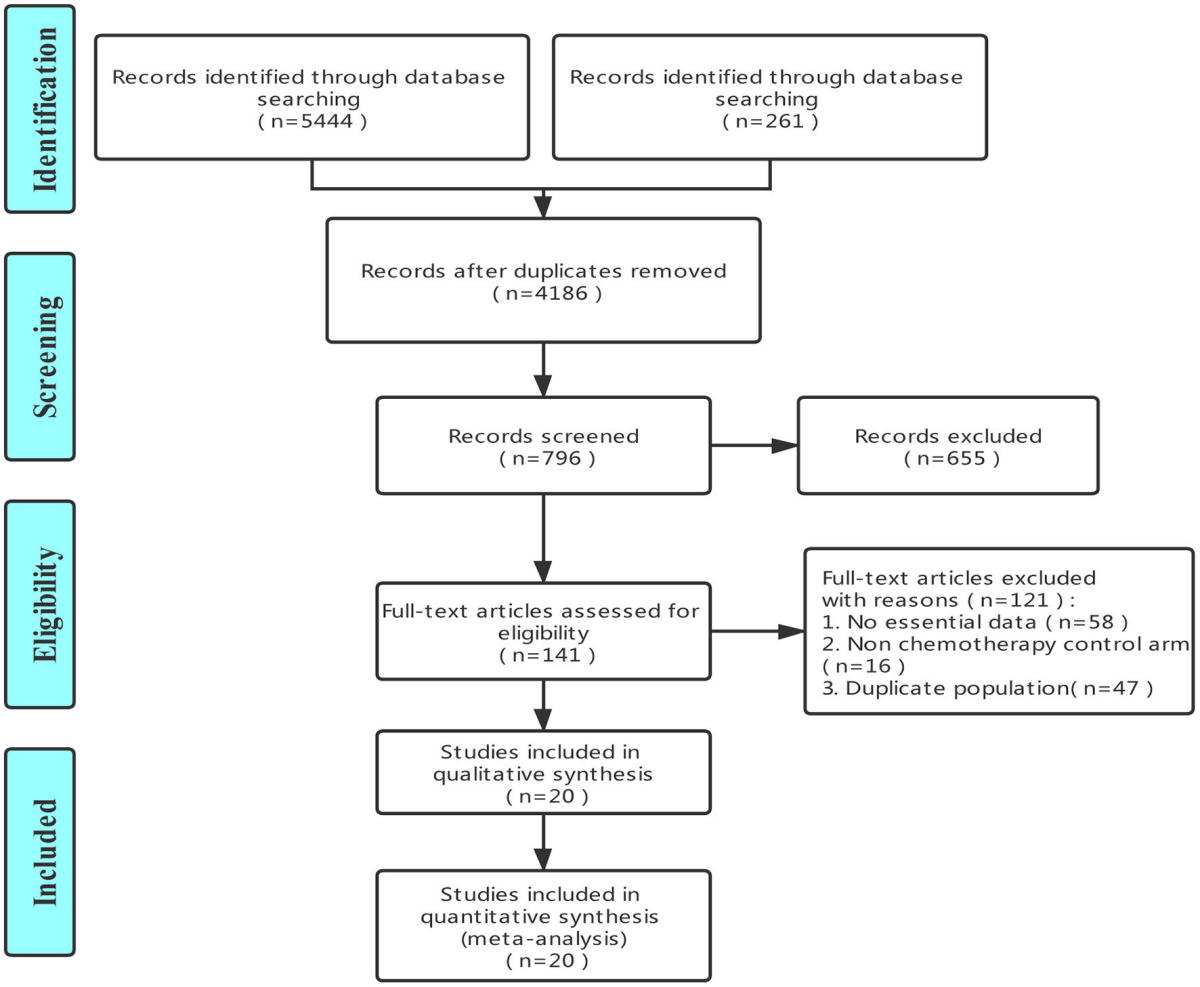
The flowchart of literature screening process.

The initial search yielded a total of 5,705 potentially eligible studies. After removing duplicates, 4,186 records were selected for the review of title and abstract. After this review, 141 studies were submitted to the assessment to full texts. Finally, upon application of the aforementioned inclusion and exclusion criteria (see *Inclusion and Exclusion Criteria*), we selected 20 clinical trials that included a total of 13,483 patients (20–39). The flowchart of the literature screening process is shown in Figure 1.

### Study Characteristics and Quality Assessment

The eligible studies included in the final dataset comprised 16 phase III clinical trials, three phase II trials, and one phase II/III trial. A total of seven trials investigated anti–PD-1 monotherapy and four trials investigated anti–PD-1 combined with chemotherapy. Furthermore, five trials investigated anti–PD-L1 monotherapy, and three trials investigated anti–PD-L1 combined with chemotherapy. Only two trials investigated anti-CTLA4 combined with chemotherapy. The detailed baseline characteristics of the trials included in the final dataset are summarized in Table 1. The results of our evaluation of methodological quality of the reviewed studies are presented in Supplementary Table 2. Most of the included trials experienced low risk, and the overall risk of bias was regarded as acceptable.

**Table 1:** The detailed baseline characteristics of included clinical trials.

**References**	**Study ID**	**NCT number**	**Phase**	**ICI drug**	**Treatment**	**Number of patients**	
						**ICI**	**Control arm**
Wu et al. (20)	CheckMate 078	NCT02613507	III	PD-1	Nivolumab	337	156
Hellmann et al. (21)	CheckMate 227(1a)	NCT02477826	III	PD-1	Nivolumab	391	387
Mok et al. (22)	KEYNOTE-042	NCT02220894	III	PD-1	Pembrolizumab	636	615
Reck et al. (23)	KEYNOTE-024	NCT02142738	III	PD-1	Pembrolizumab	154	150
Carbone et al. (24)	CheckMate 026	NCT02041533	III	PD-1	Nivolumab	267	263
Herbst et al. (25)	KEYNOTE-010	NCT01905657	II/III	PD-1	Pembrolizumab	682	309
Borghaei et al. (26)	CheckMate 057	NCT01673867	III	PD-1	Nivolumab	287	268
Brahmer et al. (27)	CheckMate 017	NCT01642004	III	PD-1	Nivolumab	131	129
Planchard et al. (28)	ARCTIC	NCT02352948	III	PD-L1	Durvalumab	179	173
Rizvi et al. (29)	MYSTIC	NCT02453282	III	PD-L1	Durvalumab	369	352
Barlesi et al. (30)	JAVELIN Lung 200	NCT02395172	III	PD-L1	Avelumab	393	365
Fehrenbacher et al. (31)	OAK	NCT02008227	III	PD-L1	Atezolizumab	609	578
Fehrenbacher et al. (32)	POPLAR	NCT01903993	II	PD-L1	Atezolizumab	142	135
Paz-Ares et al. (33)	KEYNOTE-407	NCT02775435	III	PD-1	Pembrolizumab + chemotherapy	278	280
Jotte et al. (34)	IMpower131	NCT02367794	III	PD-L1	Atezolizumab + chemotherapy	334	334
Hellmann et al. (21)	CheckMate 227(1b)	NCT02477826	III	PD-1	Nivolumab + chemotherapy	172	183
West et al. (35)	IMpower130	NCT02367781	III	PD-L1	Atezolizumab + chemotherapy	473	232
Socinski et al. (36)	IMpower150	NCT02366143	III	PD-L1	Atezolizumab + chemotherapy	393	394
Govindan et al. (37)	CA184-104	NCT01285609	III	CTLA-4	Ipilimumab + chemotherapy	388	361
Langer et al. (38)	KEYNOTE-021	NCT02039674	II	PD-1	Pembrolizumab + chemotherapy	59	62
Lynch et al. (39)	CA184-041	NCT00527735	II	CTLA-4	Ipilimumab + chemotherapy	65	71
Hellmann et al. (21)	CheckMate 227	NCT02477826	III	PD-1/CTLA4	Nivolumab + ipilimumab	576	570
Rizvi et al. (29)	MYSTIC	NCT02453282	III	PD-L1/CTLA4	Durvalumab + tremelimumab	371	352

### Incidence of Treatment-Related Fatal Adverse Events

Based on the 13,483 patients in the included trials, we investigated the incidence of treatment-related FAEs among different therapeutic strategies. As shown in Table 2, among 4,577 patients who received ICI monotherapy, the pooled incidence of FAEs was 0.65% (95% CI = 0.31–1.07, *I*^2^ = 50.2%). To compare, the pooled incidence of FAEs among 5,797 patients treated with chemotherapy was 1.17% (95% CI = 0.74–1.69, *I*^2^ = 56.3%). Furthermore, the incidence of FAEs among 947 patients who received ICI plus another ICI was 1.47% (95% CI = 0.78–2.36, *I*^2^ = 0%). Of note, the incidence of FAEs among 2,162 patients who had undergone combination therapy of ICI plus chemotherapy was 2.01% (95% CI = 1.42–2.69, *I*^2^ = 5.9%). The overall incidence of FAEs among all patients in the reviewed studies was 1.12% (95% CI = 0.83–1.45, *I*^2^ = 57.40%).

**Table 2:** Overall incidence of treatment-related fatal adverse events.

**Treatment regimens**	**No. of**	**Sample**	**Pooled**	**95% CI**	***I*^**2**^**
	**FAEs**	**size**	**incidence, %**		
ICI	38	4,577	0.65	0.31–1.07	50.2%
Chemotherapy	81	5,797	1.17	0.74–1.69	56.3%
ICI + ICI	14	947	1.47	0.78–2.36	0.00%
ICI + chemotherapy	48	2,162	2.01	1.42–2.69	5.9%
Overall	181	13,483	1.12	0.83–1.45	57.40%

### Relative Risk of Fatal Adverse Events

**Figure 2:**
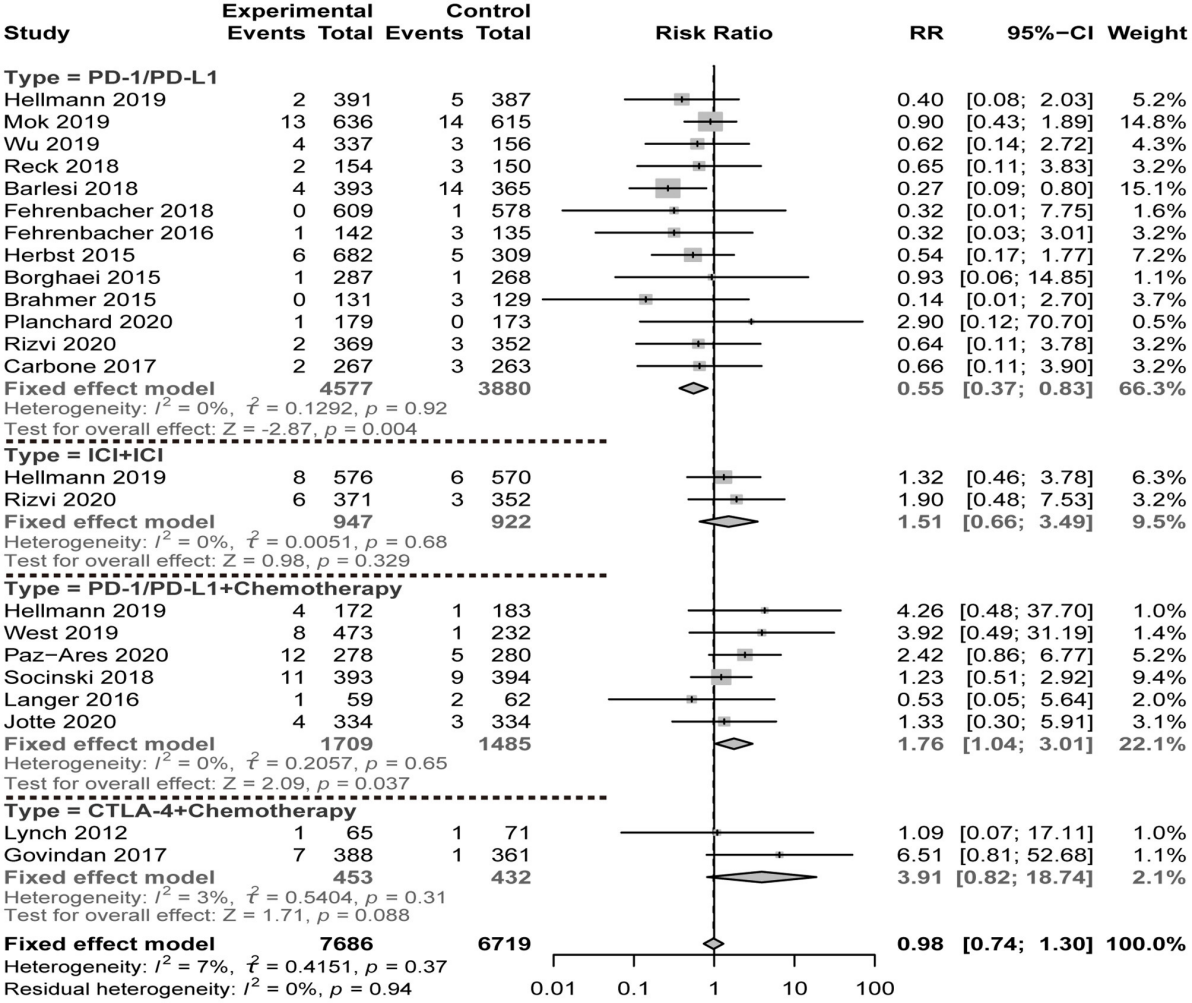
Risk of fatal adverse events of ICI therapy compared with chemotherapy.

We calculated the respective contribution of ICI monotherapy and combination therapy to the FAEs as compared to chemotherapy (Figure 2). The results showed that PD-1/PD-L1 monotherapy was significantly related to the decreased risk of FAE occurrence (RR = 0.55, 95% CI = 0.37–0.83, *P* = 0.004, *I*^2^ = 0%). In contrast, the combined therapy of PD-1/PD-L1 plus chemotherapy significantly increased the risk of FAEs (RR = 1.76, 95% CI = 1.04–3.01, *P* = 0.037, *I*^2^ = 0%). The results of our assessment of CTLA-4 plus chemotherapy revealed a similar tendency of risk (RR = 3.91, 95% CI = 0.82–18.74, *P* = 0.088, *I*^2^ = 3%). Moreover, the therapeutic regimen of ICI plus ICI also increased the risk of FAE occurrence (RR = 1.51, 95% CI = 0.66–3.49, *P* = 0.329, *I*^2^ = 0%).

### Relative Risk of System-Specific Disorders

The results of the FAE frequency classified by specific system disorders are summarized in Table 3. Compared to chemotherapy, ICI monotherapy was associated with lower incidence of FAEs caused by blood system disorders (RR = 0.23, 95% CI = 0.07–0.73, *P* = 0.013, *I*^2^ = 0%) and infectious diseases (RR = 0.29, 95% CI = 0.13–0.63, *P* = 0.002, *I*^2^ = 0%). However, no statistical differences were found in other systems. Furthermore, the results of our comparison between combination therapy and chemotherapy showed that, in the former, the percentage of almost all FAEs was higher than in the latter, although this difference did not reach statistical significance. This finding suggests a potential risk of combined therapy.

**Table 3:** Incidence and risk of system-specific FAE in ICI and chemotherapy.

	**System**	**ICI**		**Chemotherapy**		**RR**	**95% CI**	***P*-value**
		**Events/total**	**%**	**Events/total**	**%**			
Monotherapy	Infections and infestations	6/3,774	0.16	24/3,283	0.73	0.29	0.13-0.63	0.002
	Respiratory system disorders	18/3,681	0.49	12/3,034	0.40	1.17	0.59–2.34	0.656
	Blood and lymphatic system disorders	0/3,025	0	10/2,559	0.39	0.23	0.07–0.73	0.013
	Cardiac disorders	5/2,190	0.23	4/1,580	0.25	0.89	0.29–2.72	0.834
	Metabolism and nutrition disorders	0/1,711	0	3/1,289	0.23	0.25	0.04–1.54	0.136
	Renal and urinary disorders	1/393	0.25	1/365	0.27	0.93	0.06–14.79	0.958
	Nervous system disorders	2/1,314	0.15	1/1,270	0.08	1.35	0.26–6.89	0.718
	Vascular disorders	1/973	0.10	1/771	0.13	0.70	0.12–4.10	0.693
	Gastrointestinal disorders	1/636	0.16	0/615	0	2.90	0.12–71.08	0.514
	Hepatobiliary	–	–	–	–	–	–	–
	Death not otherwise specified	3/1,325	0.23	3/1,265	0.24	0.96	0.26–3.51	0.947
Combination	Infections and infestations	9/1,709	0.53	10/1,485	0.67	0.86	0.37–2.02	0.734
	Respiratory system disorders	12/1,316	0.91	5/1,089	0.46	1.99	0.75–5.26	0.165
	Gastrointestinal disorders	1/393	0.25	2/394	0.51	0.50	0.05–5.51	0.572
	Nervous system disorders	1/393	0.25	1/394	0.25	1.00	0.06–15.97	0.999
	Renal and urinary disorders	0/278	0.00	1/280	0.36	0.34	0.01–8.21	0.503
	Cardiac disorders	5/1,085	0.46	1/846	0.12	1.99	0.44–9.09	0.374
	Blood and lymphatic system disorders	4/624	0.64	1/639	0.16	2.27	0.51–10.17	0.283
	Hepatobiliary	3/1,085	0.28	0/846	0.00	2.39	0.39–14.79	0.348
	Vascular disorders	2/565	0.35	0/577	0.00	3.10	0.32–29.67	0.327
	Metabolism and nutrition disorders	—	—	—	—	—	—	—
	Death not otherwise specified	3/751	0.40	0/512	0.00	2.99	0.36-24.78	0.309

### Pooled Incidence of Specific Disease in Interested Systems

**Figure 3:**
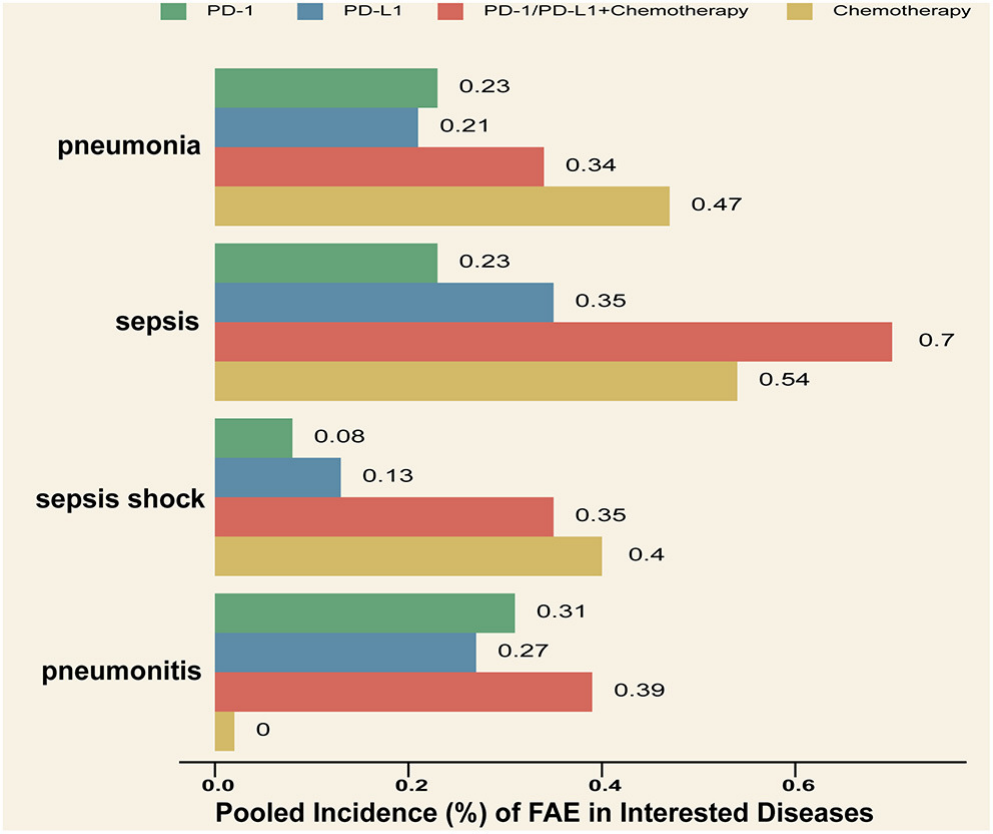
Pooled incidence of specific disease in interested systems.

In order to ensure accuracy of our meta-analysis, we initially focused on the diseases systematically reported in the reviewed studies, thus excluding isolated reports of FAEs. In the results, we noticed that the cases of FAEs in infectious diseases (49/167) and respiratory system disorders (47/167) accounted for most of the deaths among the patients from the ICI group and the ICI plus chemotherapy group (Table 3). Therefore, we further explored the incidence of FAEs in specific diseases. For infectious diseases, the pooled incidence of FAEs among the patients who received chemotherapy was remarkably higher than among those who received ICI monotherapy (Figure 3). For the respiratory system disorders, we primarily focused on the incidence of pneumonitis, which was a major cause of death among the patients who received ICI monotherapy (10/38) and was related to a potential immunologic cause. The incidence of fatal pneumonitis in ICI therapy was ~0.3% (Figure 3). Compared to chemotherapy, the application of ICI was significantly related to an increase in the incidence of pneumonitis (RR = 5.72, 95% CI = 1.14–28.80, *P* = 0.03, *I*^2^ = 0%). For other immune-related adverse events (irAEs), we did not calculate the pooled incidence because of the small numbers of cases among the reviewed studies. Definite reports of irAEs in the reviewed studies are summarized in Table 4. Taken together, the incidence of these fatal irAEs was relatively low. Among all cases, reports of hepatitis were the most frequent. Of note, except for one case of myocarditis reported after avelumab monotherapy, all other irAEs were associated with the combined therapy (ICI plus chemotherapy or ICI plus another ICI).

**Table 4:** Fatal immune-related adverse events in included NSCLC trials.

		**Events**	**Total**	**%**
Monotherapy	Myocarditis	1	393	0.254
	Hepatitis	4	1,566	0.255
Combined	Myocarditis	1	576	0.174
	Colitis	1	388	0.258

### Publication Bias

The publication bias was evaluated by the funnel plot and Egger's test. The funnel plot was symmetric (Supplementary Figure 1). The results of Egger's test also indicated the lack of publication bias (*t* = −0.10, *P* = 0.92).

## Discussion

To our knowledge, this is the largest and most comprehensive systematic review and meta-analysis that characterize FAEs associated with ICI therapy among NSCLC patients. While previous studies primarily compared safety and toxicity among various therapeutic regimens of ICIs in different cancers (13, 40), in the present review, we focused on the incidence of FAEs in different organ systems of NSCLC patients. Overall, although several previous studies reported the incidence of adverse events from different perspectives (41, 42), fatal events are relatively uncommon, and their incidence is not completely consistent with previous reports about non-fatal adverse events. The present review focused on more than 13,000 patients from 20 well-designed clinical trials. We evaluated the incidence and relative risk of FAEs in ICI therapy as compared to conventional chemotherapy. Furthermore, we also performed the pooled analysis to compare different distribution features of FAEs between immunotherapy and chemotherapy in various systems. The results demonstrated that, as compared to the patients who received standard chemotherapy, the patients who received ICI therapy had a significantly higher risk of lethal pneumonitis.

Chemotherapy remains one of the primary treatment strategies for the patients with advanced NSCLC. However, long-term chemotherapy can lead to drug resistance and side effects, which may result in poor compliance and tolerance among patients (43, 44). Immunotherapy has dramatically transformed treatment paradigms of lung cancer. Previous studies reported the safety evaluation for ICI monotherapy and combination therapy (45). Based on this evidence, in the present review, we investigated the incidence of FAEs in NSCLC patients in ICI monotherapy and combination therapy. The corresponding incidence rates were found to amount to 0.65 and 2.01%, respectively. Compared to the near 100% fatality rate for advanced lung cancer, the eligible patients could derive certain benefits from ICI therapy. However, consistently with previous studies (46), we found that the combination therapy of ICI plus chemotherapy increased the risk of FAEs as compared to standard chemotherapy. Therefore, in the selection of optimal treatment regimens, more attention should be paid to potential adverse events.

The results of our comparison of the distribution features of FAEs between ICI therapy and chemotherapy revealed that the patients who received chemotherapy were susceptible to FAEs caused by blood system disorders and infectious diseases. This significantly increased incidence of FAEs in chemotherapy can be explained by the fact that the cytotoxic drugs applied in the conventional chemotherapy regimens exert their antitumor effects via the interference to the cell cycle, thereby inducing the side effect of bone marrow suppression (47, 48). The results of our meta-analysis showed that, because of the completely different action mechanisms that make it possible to avoid the hematological toxicities and complications, ICI therapy can help to avoid the risk of myelosuppression and infectious diseases.

The trials included in the review (33, 35) have shown that ICIs combined with chemotherapy could improve the progression-free survival and overall survival. While the conventional chemotherapy can reduce the tumor burden and influence the immune regulation process, with positive synergistic effects of immunotherapy, the combination of these two regimens could result in more adverse events. In our results, the incidence of FAEs was 2.01% for the ICIs combined with chemotherapy, which was the highest among all regimens. Further explorations are needed to develop high-efficacy and low-toxicity treatment schemes.

With regard to fatal irAEs, the cases reported in the reviewed clinical trials included pneumonitis, hepatitis, myocarditis, and colitis. Incidence rate of cases with fatal irAEs was different from that of mild irAE cases with low AE grade. While fatal irAEs were rare, they tended to progress to a serious grade. Although the incidence of these fatal irAEs was ~0.2–0.3%, it requires vigilance, as the serious irAEs could disrupt the treatment scheme. Of note, the combined therapy had a potential association with fatal irAEs. Therefore, the patients who receive ICI combined therapy require increased monitoring and prompt disposal of potential irAEs, including discontinuation, supportive management, or glucocorticoids.

Our review of treatment-related FAEs suggested that pneumonitis is a frequently reported irAE in ICI arms of the reviewed clinical trials. According to several previous studies, the incidence of pneumonitis was in the range of 2–5% in all grades and 0.7–2% in grades 3–5 after ICI monotherapy (49–51). Low-grade pneumonitis could result in treatment discontinuation, and serious pneumonitis was life-threatening. The results of our meta-analysis further revealed that the incidence of fatal pneumonitis in ICI therapeutic regimens was ~0.3% in patients with advanced NSCLC. Compared to chemotherapy, ICI therapy can significantly increase the incidence of fatal pneumonitis. Accordingly, and considering that the symptoms of pneumonitis frequently lack specificity, non-specific manifestations of pneumonitis, such as progressively dry cough and shortness of breath, deserve more attention and differentiation during ICI treatment.

Finally, our results revealed the frequent causes of death in immunotherapy and chemotherapy. Specifically, while fatal events in chemotherapy were mainly derived from myelosuppression and infection, in immunotherapy, FAEs were mainly caused by non-infectious pneumonitis, which might result from the overactivation of immune system (52). These findings suggest more corresponding emphasis should be placed on potential FAEs in different antitumor pharmaceutical interventions. Disorders with potentially life-threatening risks require early detection and timely management. Particularly during the combination therapy, adverse events associated with each of the therapies can overlap and lead to fatal outcomes.

There remained several limitations in our study. First, the information on adverse events was provided by various institutions from different clinical trials. The definitions of FAE were not standardized. This might result in inaccurate data collection. Second, considering that FAEs are relatively rare, the results might be influenced by incidental events. Third, as we focused only on the RCT, the characteristics of the patients who did not meet the inclusion criteria of RCT were missing. Therefore, the results of the present review might not be generalizable to the overall population.

## Conclusions

The present review investigated the incidence of FAEs in immunotherapy among advanced NSCLC patients. Overall, compared to conventional chemotherapy, ICI monotherapy was found to decrease the risk of FAEs, while the combined therapy (with another ICI or chemotherapy) had the opposite tendency. Furthermore, while the patients who received chemotherapy mainly suffered from the risks of death from myelosuppression and infection, those who received immunotherapy were mainly threatened by immune-related pneumonitis. To conclude, our results provide meaningful insights for the assessment and management of risks associated with FAEs in the medication administration process.

## Data Availability Statement

The raw data supporting the conclusions of this article will be made available by the authors, without undue reservation.

## Author Contributions

YaZ contributed to study design, quality assessment, and revision of the manuscript. XY contributed to acquisition of data, statistical analysis, and manuscript drafting. XZ contributed to acquisition of data and quality assessment. TY and YeZ contributed to data extraction and data synthesis. All authors contributed to the article and approved the submitted version.

## Conflict of Interest

The authors declare that the research was conducted in the absence of any commercial or financial relationships that could be construed as a potential conflict of interest.

## References

[1] Siegel RL Miller KD Jemal A. Cancer statistics, 2020. CA Cancer J Clin. (2020) 70:7–30. 10.3322/caac.21590 31912902

[2] Herbst RS Morgensztern D Boshoff C. The biology and management of non-small cell lung cancer. Nature. (2018) 553:446–54. 10.1038/nature25183 29364287

[3] Aupérin A Le Péchoux C Rolland E Curran WJ Furuse K Fournel P. Meta-Analysis of concomitant versus sequential radiochemotherapy in locally advanced non-small-cell lung cancer. J Clin Oncol. (2010) 28:2181–90. 10.1200/jco.2009.26.2543 20351327

[4] Song P Zhang J Shang C Zhang L. Real-world evidence and clinical observations of the treatment of advanced non-small cell lung cancer with PD-1/PD-L1 inhibitors. Sci Rep. (2019) 9:4278. 10.1038/s41598-019-40748-7 30862891 PMC6414649

[5] Sibiya M Raphoko L Mangokoana D Makola R Nxumalo W Matsebatlela T. Induction of cell death in human A549 cells using 3-(Quinoxaline-3-yl) Prop-2-ynyl methanosulphonate and 3-(Quinoxaline-3-yl) Prop-2-yn-1-ol. Molecules. (2019) 24:407. 10.3390/molecules24030407 30678061 PMC6384999

[6] Guibert N Hu Y Feeney N Kuang Y Plagnol V Jones G. Amplicon-based next-generation sequencing of plasma cell-free DNA for detection of driver and resistance mutations in advanced non-small cell lung cancer. Ann Oncol. (2018) 29:1049–55. 10.1093/annonc/mdy005 29325035 PMC5913609

[7] Lee YT Tan YJ Oon CE. Molecular targeted therapy: treating cancer with specificity. Eur J Pharmacol. (2018) 834:188–96. 10.1016/j.ejphar.2018.07.034 30031797

[8] Onoi K Chihara Y Uchino J Shimamoto T Morimoto Y Iwasaku M. Immune checkpoint inhibitors for lung cancer treatment: a review. J Clin Med. (2020) 9:1362. 10.3390/jcm9051362 32384677 PMC7290914

[9] Lantuejoul S Damotte D Hofman V Adam J. Programmed death ligand 1 immunohistochemistry in non-small cell lung carcinoma. J Thorac Dis. (2019) 11:S89–101. 10.21037/jtd.2018.12.103 30775032 PMC6353738

[10] Pardoll DM. The blockade of immune checkpoints in cancer immunotherapy. Nat Rev Cancer. (2012) 12:252–64. 10.1038/nrc3239 22437870 PMC4856023

[11] Huang Z Su W Lu T Wang Y Dong Y Qin Y. First-line immune-checkpoint inhibitors in non-small cell lung cancer: current landscape and future progress. Front Pharmacol. (2020) 11:578091. 10.3389/fphar.2020.578091 33117170 PMC7577011

[12] Darvin P Toor SM Sasidharan Nair V Elkord E. Immune checkpoint inhibitors: recent progress and potential biomarkers. Exp Mol Med. (2018) 50:165. 10.1038/s12276-018-0191-1 30546008 PMC6292890

[13] Wang DY Salem J-E Cohen JV Chandra S Menzer C Ye F. Fatal Toxic effects associated with immune checkpoint inhibitors. JAMA Oncol. (2018) 4:1721–8. 10.1001/jamaoncol.2018.3923 30242316 PMC6440712

[14] Chang C-Y Park H Malone DC Wang C-Y Wilson DL Yeh Y-M. Immune checkpoint inhibitors and immune-related adverse events in patients with advanced melanoma. JAMA Netw Open. (2020) 3:e201611. 10.1001/jamanetworkopen.2020.1611 32211869 PMC7097702

[15] Petrelli F Grizzi G Ghidini M Ghidini A Ratti M Panni S. Immune-related adverse events and survival in solid tumors treated with immune checkpoint inhibitors: a systematic review and meta-analysis. J Immunother. (2020) 43:1–7. 10.1097/CJI.0000000000000300 31574022

[16] Roberts J Ennis D Hudson M Ye C Saltman A Himmel M. Rheumatic immune-related adverse events associated with cancer immunotherapy: a nationwide multi-center cohort. Autoimmun Rev. (2020) 19:102595. 10.1016/j.autrev.2020.102595 32535092

[17] Herrmann SM Perazella MA. Immune checkpoint inhibitors and immune-related adverse renal events. Kidney Int Rep. (2020) 5:1139–48. 10.1016/j.ekir.2020.04.018 32775813 PMC7403510

[18] Moher D Liberati A Tetzlaff J Altman DG. Preferred reporting items for systematic reviews and meta-analyses: the PRISMA statement. BMJ. (2009) 339:b2535. 10.1136/bmj.b2535 19622551 PMC2714657

[19] Higgins JPT Altman DG Gotzsche PC Juni P Moher D Oxman AD. The cochrane collaboration's tool for assessing risk of bias in randomised trials. BMJ. (2011) 343:d5928. 10.1136/bmj.d5928 22008217 PMC3196245

[20] Wu Y-L Lu S Cheng Y Zhou C Wang J Mok T. Nivolumab versus docetaxel in a predominantly chinese patient population with previously treated advanced NSCLC: CheckMate 078 randomized phase III clinical trial. J Thorac Oncol. (2019) 14:867–75. 10.1016/j.jtho.2019.01.006 30659987

[21] Hellmann MD Paz-Ares L Bernabe Caro R Zurawski B Kim S-W Carcereny Costa E. Nivolumab plus ipilimumab in advanced non-small-cell lung cancer. N Engl J Med. (2019) 381:2020–31. 10.1056/NEJMoa1910231 31562796

[22] Mok TSK Wu YL Kudaba I Kowalski DM Cho BC Turna HZ. Pembrolizumab versus chemotherapy for previously untreated, PD-L1-expressing, locally advanced or metastatic non-small-cell lung cancer (KEYNOTE-042): a randomised, open-label, controlled, phase 3 trial. Lancet. (2019) 393:1819–30. 10.1016/S0140-6736(18)32409-7 30955977

[23] Reck M Rodríguez-Abreu D Robinson AG Hui R Csoszi T Fülöp A. Updated analysis of KEYNOTE-024: pembrolizumab versus platinum-based chemotherapy for advanced non-small-cell lung cancer with PD-L1 tumor proportion score of 50% or greater. J Clin Oncol. (2019) 37:537–46. 10.1200/JCO.18.00149 30620668

[24] Carbone DP Reck M Paz-Ares L Creelan B Horn L Steins M. First-line nivolumab in stage iv or recurrent non-small-cell lung cancer. N Engl J Med. (2017) 376:2415–26. 10.1056/NEJMoa1613493 28636851 PMC6487310

[25] Herbst RS Baas P Kim D-W Felip E Pérez-Gracia JL Han J-Y. Pembrolizumab versus docetaxel for previously treated, PD-L1-positive, advanced non-small-cell lung cancer (KEYNOTE-010): a randomised controlled trial. Lancet. (2016) 387:1540–50. 10.1016/s0140-6736(15)01281-7 26712084

[26] Borghaei H Paz-Ares L Horn L Spigel DR Steins M Ready NE. Nivolumab versus docetaxel in advanced nonsquamous non-small-cell lung cancer. N Engl J Med. (2015) 373:1627–39. 10.1056/NEJMoa1507643 26412456 PMC5705936

[27] Brahmer J Reckamp KL Baas P Crinò L Eberhardt WEE Poddubskaya E. Nivolumab versus docetaxel in advanced squamous-cell non-small-cell lung cancer. N Engl J Med. (2015) 373:123–35. 10.1056/NEJMoa1504627 26028407 PMC4681400

[28] Planchard D Reinmuth N Orlov S Fischer JR Sugawara S Mandziuk S. ARCTIC: durvalumab with or without tremelimumab as third-line or later treatment of metastatic non-small-cell lung cancer. Ann Oncol. (2020) 31:609–18. 10.1016/j.annonc.2020.02.006 32201234

[29] Rizvi NA Cho BC Reinmuth N Lee KH Luft A Ahn M-J. Durvalumab With or without tremelimumab vs standard chemotherapy in first-line treatment of metastatic non-small cell lung cancer. JAMA Oncol. (2020) 6:661–74. 10.1001/jamaoncol.2020.0237 32271377 PMC7146551

[30] Barlesi F Vansteenkiste J Spigel D Ishii H Garassino M de Marinis F. Avelumab versus docetaxel in patients with platinum-treated advanced non-small-cell lung cancer (JAVELIN Lung 200): an open-label, randomised, phase 3 study. Lancet Oncol. (2018) 19:1468–79. 10.1016/s1470-2045(18)30673-9 30262187

[31] Fehrenbacher L von Pawel J Park K Rittmeyer A Gandara DR Ponce Aix S. Updated efficacy analysis including secondary population results for OAK: a randomized phase III Study of atezolizumab versus docetaxel in patients with previously treated advanced non-small cell lung cancer. J Thorac Oncol. (2018) 13:1156–70. 10.1016/j.jtho.2018.04.039 29777823

[32] Fehrenbacher L Spira A Ballinger M Kowanetz M Vansteenkiste J Mazieres J. Atezolizumab versus docetaxel for patients with previously treated non-small-cell lung cancer (POPLAR): a multicentre, open-label, phase 2 randomised controlled trial. Lancet. (2016) 387:1837–46. 10.1016/s0140-6736(16)00587-0 26970723

[33] Paz-Ares L Vicente D Tafreshi A Robinson A Soto Parra H Mazières J. A randomized, placebo-controlled trial of pembrolizumab plus chemotherapy in patients with metastatic squamous NSCLC: protocol-specified final analysis of KEYNOTE-407. J Thorac Oncol. (2020) 15:1657–69. 10.1016/j.jtho.2020.06.015 32599071

[34] Jotte R Cappuzzo F Vynnychenko I Stroyakovskiy D Rodríguez-Abreu D Hussein M. Atezolizumab in combination with carboplatin and nab-paclitaxel in advanced squamous NSCLC (IMpower131): results from a randomized phase III trial. J Thorac Oncol. (2020) 15:1351–60. 10.1016/j.jtho.2020.03.028 32302702

[35] West H McCleod M Hussein M Morabito A Rittmeyer A Conter HJ. Atezolizumab in combination with carboplatin plus nab-paclitaxel chemotherapy compared with chemotherapy alone as first-line treatment for metastatic non-squamous non-small-cell lung cancer (IMpower130): a multicentre, randomised, open-label, phase 3 trial. Lancet Oncol. (2019) 20:924–37. 10.1016/s1470-2045(19)30167-6 31122901

[36] Socinski MA Jotte RM Cappuzzo F Orlandi F Stroyakovskiy D Nogami N. Atezolizumab for first-line treatment of metastatic non-squamous NSCLC. N Engl J Med. (2018) 378:2288–301. 10.1056/NEJMoa1716948 29863955

[37] Govindan R Szczesna A Ahn MJ Schneider CP Gonzalez Mella PF Barlesi F. Phase III trial of ipilimumab combined with paclitaxel and carboplatin in advanced squamous non-small-cell lung cancer. J Clin Oncol. (2017) 35:3449–57. 10.1200/JCO.2016.71.7629 28854067

[38] Langer CJ Gadgeel SM Borghaei H Papadimitrakopoulou VA Patnaik A Powell SF. Carboplatin and pemetrexed with or without pembrolizumab for advanced, non-squamous non-small-cell lung cancer: a randomised, phase 2 cohort of the open-label KEYNOTE-021 study. Lancet Oncol. (2016) 17:1497–508. 10.1016/s1470-2045(16)30498-3 27745820 PMC6886237

[39] Lynch TJ Bondarenko I Luft A Serwatowski P Barlesi F Chacko R. Ipilimumab in combination with paclitaxel and carboplatin as first-line treatment in stage IIIB/IV non-small-cell lung cancer: results from a randomized, double-blind, multicenter phase II study. J Clin Oncol. (2012) 30:2046–54. 10.1200/jco.2011.38.4032 22547592

[40] Liu T Jin B Chen J Wang H Lin S Dang J. Comparative risk of serious and fatal treatment-related adverse events caused by 19 immune checkpoint inhibitors used in cancer treatment: a network meta-analysis. Ther Adv Med Oncol. (2020) 12:1–25. 10.1177/1758835920940927 32774474 PMC7394035

[41] Chen R Hou X Yang L Zhao D. Comparative efficacy and safety of first-line treatments for advanced non-small cell lung cancer with immune checkpoint inhibitors: a systematic review and meta-analysis. Thorac Cancer. (2019) 10:607–23. 10.1111/1759-7714.12971 30734504 PMC6449246

[42] Lin LL Lin GF Yang F Chen XQ. A systematic review and meta-analysis of immune-mediated liver dysfunction in non-small cell lung cancer. Int Immunopharmacol. (2020) 83:106537. 10.1016/j.intimp.2020.106537 32371246

[43] Oun R Moussa YE Wheate NJ. The side effects of platinum-based chemotherapy drugs: a review for chemists. Dalton Trans. (2018) 47:6645–53. 10.1039/c8dt00838h 29632935

[44] Xu C Chen Y-P Du X-J Liu J-Q Huang C-L Chen L. Comparative safety of immune checkpoint inhibitors in cancer: systematic review and network meta-analysis. BMJ. (2018) 363:k4226. 10.1136/bmj.k4226 30409774 PMC6222274

[45] Wu Y Shi H Jiang M Qiu M Jia K Cao T. The clinical value of combination of immune checkpoint inhibitors in cancer patients: a meta-analysis of efficacy and safety. Int J Cancer. (2017) 141:2562–70. 10.1002/ijc.31012 28833119

[46] Zhou Y Chen C Zhang X Fu S Xue C Ma Y. Immune-checkpoint inhibitor plus chemotherapy versus conventional chemotherapy for first-line treatment in advanced non-small cell lung carcinoma: a systematic review and meta-analysis. J Immunother Cancer. (2018) 6:155. 10.1186/s40425-018-0477-9 30577837 PMC6303974

[47] Fu H Gao H Qi X Zhao L Wu D Bai Y. Aldolase A promotes proliferation and G_1_/S transition via the EGFR/MAPK pathway in non-small cell lung cancer. Cancer Commun. (2018) 38:18. 10.1186/s40880-018-0290-3 29764507 PMC5993145

[48] Sah SK Karn A Shah A Paudel BD Adhikari K Acharya B. Incidence and attributes of chemotherapy induced myelotoxicity, anemia and neutropenia in adults with cancer in Nepal: a cross-sectional observational study. J Oncol Pharm Pract. (2019) 25:1823–30. 10.1177/1078155218817815 30537917

[49] Da L Teng Y Wang N Zaguirre K Liu Y Qi Y. Organ-specific immune-related adverse events associated with immune checkpoint inhibitor monotherapy versus combination therapy in cancer: a meta-analysis of randomized controlled trials. Front Pharmacol. (2020) 10:1671. 10.3389/fphar.2019.01671 32082164 PMC7002539

[50] Li W Tian P Jiang Y Ren P Wang C Shao J. Treatment- and immune-related adverse events of immune checkpoint inhibitors in advanced lung cancer. Biosci Rep. (2020) 40:BSR20192347. 10.1042/bsr20192347 32315071 PMC7953488

[51] Cadranel J Canellas A Matton L Darrason M Parrot A Naccache J-M. Pulmonary complications of immune checkpoint inhibitors in patients with nonsmall cell lung cancer. Eur Respir Rev. (2019) 28:190058. 10.1183/16000617.0058-2019 31597674 PMC9488121

[52] Choi J Lee SY. Clinical characteristics and treatment of immune-related adverse events of immune checkpoint inhibitors. Immune Netw. (2020) 20:e9. 10.4110/in.2020.20.e9 32158597 PMC7049586

