# Supplementary material for: Fatal Adverse Events Associated With Immune Checkpoint Inhibitors in Non–small Cell Lung Cancer: A Systematic Review and Meta-Analysis
Source: Front Med (Lausanne). 2021 Feb 15;8:627089. doi: 10.3389/fmed.2021.627089 (PMC7917063; doi:10.3389/fmed.2021.627089)
Supplement: Supplementary file 1 [file Data_Sheet_1.DOCX]

**Supplementary materials**


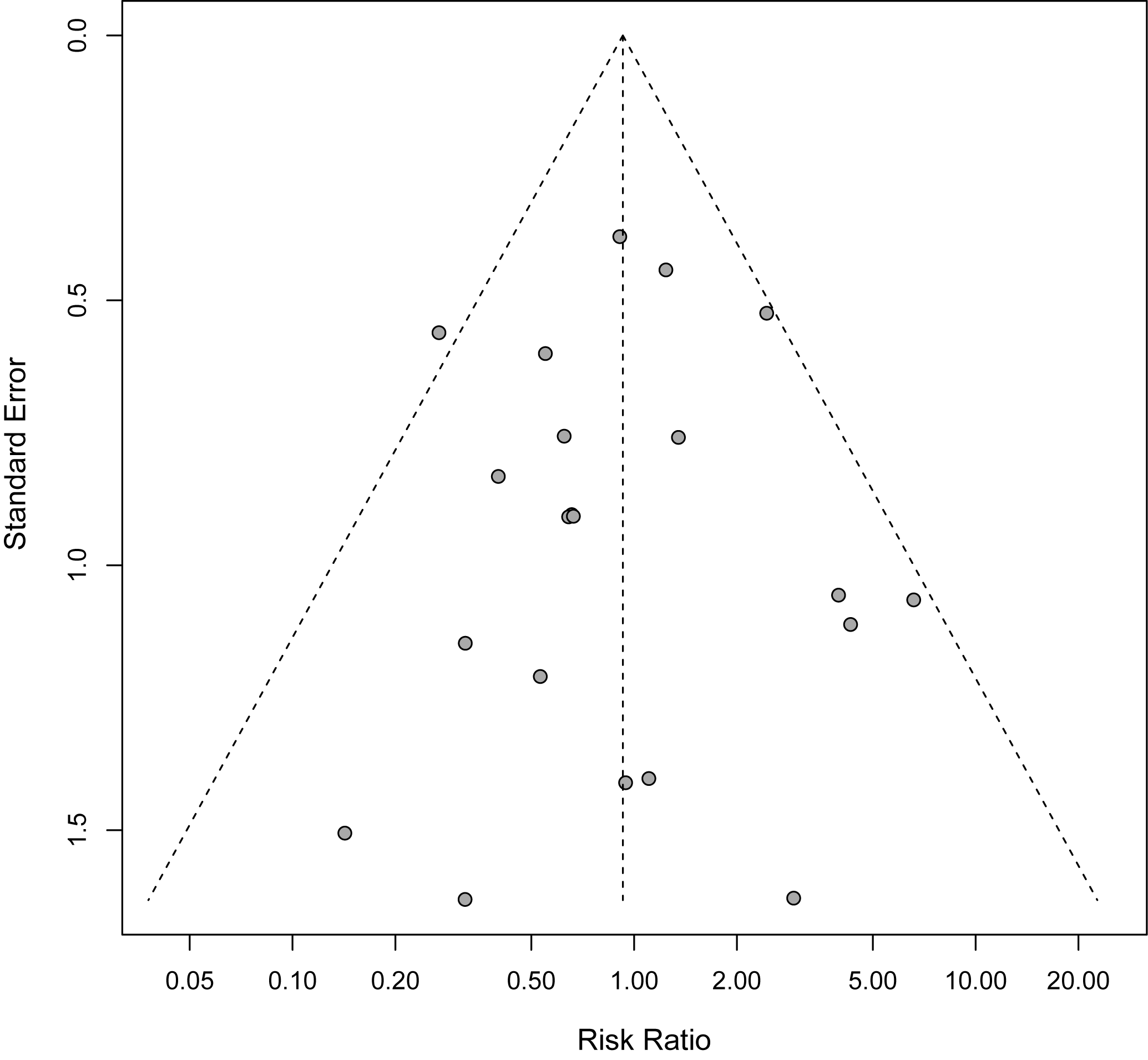


**Supplementary Figure S1** The funnel plot for fatal adverse events in patients treated with ICIs compared with chemotherapy.

**Supplementary Table S1** The details of the search strategy.

| **Pubmed** |
| --- |
| ((((((((((((Nivolumab[MeSH Terms]) OR (nivolumab)) OR (Opdivo)) OR (BMS 936558)) OR (BMS-936558)) OR (GTPL7335)) OR (MDX 1106))  OR (MDX-1106)) OR (ONO 4538)) OR (ONO-4538)) OR  ((((((((((Pembrolizumab[MeSH Terms]) OR (pembrolizumab)) OR (Keytruda)) OR (Lambrolizumab)) OR (Merck 3475)) OR (MK 3475))  OR (MK-3475)) OR (MK3475)) OR (Sch 900475)) OR (SCH-900475)) OR  ((((((Programmed Cell Death 1 Receptor[MeSH Terms]) OR (PD-1)) OR (PD 1)) OR (PD 1 Receptor)) OR (PD-1 Receptor)) OR (CD279 Antigen)))  OR  (((((((((Atezolizumab[MeSH Terms]) OR (atezolizumab)) OR (Tecentriq)) OR (MPDL 3280A)) OR (MPDL-3280A)) OR (MPDL3280A)) OR (RG-7446)) OR (RG7446)) OR  ((((((Durvalumab[MeSH Terms]) OR (durvalumab)) OR (Imfinzi)) OR (MEDI 4736)) OR (MEDI-4736)) OR (MEDI4736)) OR  (((((((Avelumab[MeSH Terms]) OR (avelumab)) OR (Bavencio)) OR (MSB-0010682)) OR (MSB0010682)) OR (MSB0010718C)) OR (MSB-0010718C)) OR  ((((((B7-H1 Antigen[MeSH Terms]) OR (PD-L1)) OR (PDL1)) OR (PD L1)) OR (Programmed Cell Death 1 Ligand 1 Protein)) OR (CD274 Antigens)))  OR  (((((((((Ipilimumab[MeSH Terms]) OR (ipilimumab)) OR (Yervoy)) OR (BMS-734016)) OR (MDX-010)) OR (MDX-101)) OR (MDX-CTLA-4)) OR (MOAB-CTLA-4)) OR  (((((((Tremelimumab[MeSH Terms]) OR (tremelimumab)) OR (Ticilimumab)) OR (CP675 cpd)) OR (CP-675)) OR (CP-675,206)) OR (CP-675206)) OR  ((((((CTLA-4 Antigen[MeSH Terms]) OR (CTLA-4)) OR (CTLA 4)) OR (Cytotoxic T-Lymphocyte Associated Antigen 4))  OR (Cytotoxic T-Lymphocyte Antigen 4)) OR (CD152 Antigen)))  OR  (immune checkpoint inhibitor*))  AND ((Carcinoma, Non-Small-Cell Lung[MeSH Terms]) OR (NSCLC) OR (non-small cell lung cancer)) |
| **Embase** |
| #1 'non small cell lung cancer'/exp  #2 NSCLC  #3 #1 OR #2  #4 'pembrolizumab'/exp  #5 'pembrolizumab'  #6 keytruda  #7 'lambrolizumab'  #8 'Merck 3475' OR 'MK 3475' OR 'MK-3475' OR 'MK3475' OR 'Sch 900475' OR 'SCH-900475'  #9 'nivolumab'/exp  #10 'nivolumab'  #11 opdivo  #12 'BMS 936558' OR 'BMS-936558' OR 'GTPL7335' OR 'MDX 1106' OR 'MDX-1106' OR 'ONO 4538' OR 'ONO-4538'  #13 'programmed death 1 receptor'/exp  #14 'Programmed Cell Death 1 Receptor' OR 'PD-1' OR 'PD 1' OR 'PD 1 Receptor' OR 'PD-1 Receptor' OR 'CD279 Antigen'  #15 'atezolizumab'/exp  #16 'atezolizumab'  #17 tecentriq  #18 'MPDL 3280A' OR 'MPDL-3280A' OR 'MPDL3280A' OR 'RG-7446' OR 'RG7446'  #19 'durvalumab'/exp  #20 'durvalumab'  #21 imfinzi  #22 'MEDI 4736' OR 'MEDI-4736' OR 'MEDI4736'  #23 'avelumab'/exp  #24 'avelumab'  #25 bavencio  #26 'MSB-0010682' OR 'MSB0010682' OR 'MSB0010718C' OR 'MSB-0010718C'  #27 'programmed death 1 ligand 1'/exp  #28 'PD-L1' OR 'PDL1' OR 'PD L1' OR 'Programmed Cell Death 1 Ligand 1 Protein' OR 'CD274 Antigens'  #29 'ipilimumab'/exp  #30 'ipilimumab'  #31 yervoy  #32 'BMS-734016' OR 'MDX-010' OR 'MDX-101' OR 'MDX-CTLA-4' OR 'MOAB-CTLA-4'  #33 'tremelimumab'/exp  #34 'tremelimumab'  #35 'ticilimumab'  #36 'CP675 cpd' OR 'CP-675' OR 'CP-675,206' OR 'CP-675206'  #37 'cytotoxic T lymphocyte antigen 4'/exp  #38 'CTLA-4 Antigen' OR 'CTLA-4' OR 'CTLA 4' OR 'Cytotoxic T-Lymphocyte Associated Antigen 4' OR 'Cytotoxic T-Lymphocyte Antigen 4' OR 'CD152 Antigen'  #39 'immune checkpoint inhibitor*'  #40 #4 OR #5 OR #6 OR #7 OR #8 OR #9 OR #10 OR #11 OR #12 OR #13 OR #14 OR #15 OR #16 OR #17 OR #18 OR #19 OR #20 OR #21 OR #22 OR #23 OR #24 OR #25 OR #26 OR #27 OR #28 OR #29 OR #30 OR #31 OR #32 OR #33 OR #34 OR #35 OR #36 OR #37 OR #38 OR #39  #41 #3 AND #40 |
| **Cochrane Library** |
| #1 MeSH descriptor: [Carcinoma, Non-Small-Cell Lung] explode all trees  #2 non-small cell lung cancer  #3 NSCLC  #4 #1 OR #2 OR #3  #5 MeSH descriptor: [Nivolumab] explode all trees  #6 Nivolumab  #7 Opdivo  #8 BMS 936558  #9 BMS-936558  #10 GTPL7335  #11 MDX 1106  #12 MDX-1106  #13 ONO 4538  #14 ONO-4538  #15 #5 OR #6 OR #7 OR #8 OR #9 OR #10 OR #11 OR #12 OR #13 OR #14  #16 Pembrolizumab  #17 Keytruda  #18 Lambrolizumab  #19 Merck 3475  #20 MK 3475  #21 MK-3475  #22 MK3475  #23 Sch 900475  #24 SCH-900475  #25 #16 OR #17 OR #18 OR #19 OR #20 OR #21 OR #22 OR #23 OR #24  #26 MeSH descriptor: [Programmed Cell Death 1 Receptor] explode all trees  #27 Programmed Cell Death 1 Receptor  #28 PD-1  #29 PD 1  #30 PD 1 Receptor  #31 PD-1 Receptor  #32 CD279 Antigen  #33 #26 OR #27 OR #28 OR #29 OR #30 OR #31 OR #32  #34 Atezolizumab  #35 Tecentriq  #36 MPDL 3280A  #37 MPDL-3280A  #38 MPDL3280A  #39 RG-7446  #40 RG7446  #41 #34 OR #35 OR #36 OR #37 OR #38 OR #39 OR #40  #42 Durvalumab  #43 Imfinzi  #44 MEDI 4736  #45 MEDI-4736  #46 MEDI4736  #47 #42 OR #43 OR #44 OR #45 OR #46  #48 Avelumab  #49 Bavencio  #50 MSB-0010682  #51 MSB0010682  #52 MSB0010718C  #53 MSB-0010718C  #54 #48 OR #49 OR #50 OR #51 OR #52 OR #53  #55 MeSH descriptor: [B7-H1 Antigen] explode all trees  #56 PD-L1  #57 PDL1  #58 PD L1  #59 Programmed Cell Death 1 Ligand 1 Protein  #60 CD274 Antigens  #61 #55 OR #56 OR #57 OR #58 OR #59 OR #60  #62 MeSH descriptor: [Ipilimumab] explode all trees  #63 Ipilimumab  #64 Yervoy  #65 BMS-734016  #66 MDX-010  #67 MDX-101  #68 MDX-CTLA-4  #69 MOAB-CTLA-4  #70 #62 OR #63 OR #64 OR #65 OR #66 OR #67 OR #68 OR #69  #71 Tremelimumab  #72 Ticilimumab  #73 CP675 cpd  #74 CP-675  #75 CP-675,206  #76 CP-675206  #77 #71 OR #72 OR #73 OR #74 OR #75 OR #76  #78 MeSH descriptor: [CTLA-4 Antigen] explode all trees  #79 CTLA-4 Antigen  #80 CTLA-4  #81 CTLA 4  #82 Cytotoxic T-Lymphocyte Associated Antigen 4  #83 Cytotoxic T-Lymphocyte Antigen 4  #84 CD152 Antigen  #85 #78 OR #79 OR #80 OR #81 OR #82 OR #83 OR #84  #86 immune checkpoint inhibitor*  #87 #15 OR #25 OR #33 OR #41 OR #47 OR #54 OR #61 OR #70 OR #77 OR #85 OR #86  #88 #4 AND #87 |

**Supplementary Table S2** The methodological quality assessment to 20 included trials.

| First author | Study ID | Random Sequence Generation | Allocation Concealment | Blinding of Participants and Personnel | Blinding of Outcome Assessment | Incomplete Outcome Data | Selective Reporting | Other Bias |
| --- | --- | --- | --- | --- | --- | --- | --- | --- |
| Hellmann 2019 | CheckMate 227 | low | low | high | low | low | low | low |
| Mok 2019 | KEYNOTE-042 | low | low | high | unclear | low | low | low |
| Wu 2019 | CheckMate 078 | low | low | high | low | low | low | low |
| Reck 2018 | KEYNOTE-024 | low | low | high | low | low | low | low |
| Barlesi 2018 | JAVELIN Lung 200 | low | low | high | low | low | low | low |
| Fehrenbacher 2018 | OAK | low | low | unclear | unclear | low | low | low |
| Fehrenbacher 2016 | POPLAR | low | low | high | low | low | low | low |
| Herbst 2015 | KEYNOTE-010 | low | low | high | unclear | low | low | low |
| Borghaei 2015 | CheckMate 057 | low | low | high | unclear | low | low | low |
| Brahmer 2015 | CheckMate 017 | low | low | high | low | low | low | low |
| Planchard 2020 | ARCTIC | low | low | high | low | low | low | low |
| Rizvi 2020 | MYSTIC | low | low | high | low | low | low | low |
| Carbone 2017 | CheckMate 026 | low | low | high | unclear | low | low | low |
| West 2019 | IMpower130 | low | low | high | low | low | low | low |
| Paz-Ares 2020 | KEYNOTE-407 | low | low | low | low | low | low | low |
| Socinski 2018 | IMpower150 | low | low | high | unclear | low | low | low |
| Langer 2016 | KEYNOTE-021 | low | low | high | low | low | low | low |
| Lynch 2012 | CA184-041 | low | low | low | low | low | low | low |
| Jotte 2020 | IMpower131 | low | low | high | low | low | low | low |
| Govindan 2017 | CA184-104 | low | low | low | low | low | low | low |
